# Remarkable Case of Triple Dentition

**Published:** 1844-09

**Authors:** 


*Remarkable case of Triple Dentition.*—Elizabeth Morelli, who was a well formed woman, had enjoyed good health, and had never suffered any inconvenience, except from a few attacks of tooth-ache, which occurred about the middle of March, 1821. She was induced, from the severity of the pain, to have the two left molar teeth pulled out. Toward the end of October in the same year, she felt some very acute pain in the part from which the teeth had been removed; and soon after two new teeth made their appearance. In January, 1826, the new teeth became loose, and causing considerable pain, she had them also drawn out. These teeth were very white and well-shaped, without the least appearance of caries. On the 16th of July, in the same year, the patient consulted Signor Aimonino, complaining of intolerable pain in the same part of the jaw as before. General and local antiphlogistic measures were tried without avail, and on the 18th, the patient perceived that two teeth of the same figure and size as the former had again made their appearance: these teeth were seen by Aimonino, so that there can be no doubt of the accuracy of the report of the case.

Although this has been called a case of triple dentition, it might with greater propriety have been called a *quadruple* dentition, since the temporary teeth are not included in the history of the case.—*Annali Univers. Di Medicina di Milano.—Forceps.*

